# Responding to “Terminal anorexia nervosa: three cases and proposed clinical characteristics”

**DOI:** 10.1186/s40337-022-00612-y

**Published:** 2022-06-25

**Authors:** Rebekah A. Mack, Caroline E. Stanton

**Affiliations:** 1 The Johns Hopkins Hospital, 600 N. Wolfe Street, Baltimore, MD 21287 USA; 2 The Medical University of South Carolina, 151 Rutledge Ave, Charleston, SC 29425 USA

**Keywords:** Eating disorders, Anorexia nervosa, Medical aid in dying, Suicide

## Abstract

The treatment of eating disorders raises many ethical debates given the pervasiveness with which this illness impacts individuals, especially as the length of time with the illness increases. A recent case study supported the appropriateness of pursuing medical aid in dying for individuals with eating disorders who wish to end their fight with their disorder. This correspondence raises concerns related to this controversial proposal as the current authors dispute that the use of medical aid in dying for individuals with eating disorders is ethically judicious or appropriate. Additionally, this correspondence highlights additional treatment implications that should be considered when trying to provide individuals with eating disorders with the best evidence-based care possible, with the goal of promoting steps toward recovery.

## Main text

The case report conducted by Gaudiani et al. [1], describing clinical considerations regarding medical aid in dying (MAID) in cases of “terminal” anorexia nervosa (AN), initiates a conversation rift with controversy regarding the ethical treatment for individuals with eating disorders (EDs). While it may be appropriate to support the compassionate, palliative approach for those individuals who have decided they no longer wish to actively seek treatment, this decision should not be entered into lightly, nor should it be accompanied by the use of MAID, even when legal in the state. Gaudiani’s article [1] highlights this as an appropriate intervention for individuals who are experiencing “terminal” AN, albeit this criterion is yet to be validated [2].

Within Jessica and Alyssa’s cases from Gaudiani’s article, determination of their illnesses as terminal, and thus the provision of MAID, was grounded in their presumed prognosis of 6 months or less to live [1]. While literature supports that refusal of nourishment will likely lead to death within 6 months [3], using this timeframe as an indicator of terminal illness neglects to consider that effective ED treatment exists and that individuals can recover even after enduring many trials of treatment and battling the disorder for extensive lengths of time [4, 5]. Additionally, the use of 6 months as a criterion for terminal illness is not substantially validated within literature. According to Hui et al.’s systematic review, nine out of the eleven definitions for a terminal illness evaluated defined the associated life expectancy with as much as “24 months or less” and as little as “days or weeks” [2, p. 86]. Characterizing a patient’s diagnosis of AN as terminal is also problematic given that the mechanism of inevitable death is the refusal to eat, yet this is a treatable symptom of the disorder [6]. Though it is recognized that undergoing ED treatment is oftentimes psychologically challenging, the ability to sustain life through medical intervention differentiates AN from other conditions which may be considered terminal, such as end-stage liver disease or cardiac failure, in which the body becomes “physiologically resistant” to medical intervention [6, p. 34]. Furthermore, labeling the illness as terminal likely decreases one’s hope that their illness can improve, with hope being a key facilitator of recovery [7]. By providing patients with access to MAID, healthcare professionals are supporting those individuals in taking an active step toward death, with limited evidence that the person with an ED is truly terminally ill. Although not mutually agreed upon by suicidologists, some consider MAID to be a form of suicide (self-killer; self-destroyer) [8] as MAID is a tool utilized to self-inflict death. As stated by the American Medical Association, “the physician provides sleeping pills and information about the lethal dose, while aware that the patient may commit suicide” [9]. In contrast, cessation of treatment is a passive decision toward death—an allowance of the inevitable that will impact every human being at some point.

Several concerns arise throughout Gaudiani’s article: first, is the proposal of “age of 30 or older” as a criterion for terminal AN [1, p. 30]. While EDs are oftentimes stereotyped as solely impacting teenage White women, they impact individuals of all sexes, genders, ages, ethnicities, races, and socioeconomic statuses [10, 11]. Although Gaudiani does not state that individuals of “older age” cannot recover, this is a commonly held myth which would be perpetuated by the inclusion of the age of 30 as a criterion for terminal AN. Notably, a study which included a sample size of 5,839 individuals with EDs found that the mean age at index admission was 35.5 years [4]. Research indicates that it is possible for these individuals of “older age” to attain recovery. According to data from a longitudinal study [5], approximately two-thirds of individuals with AN and bulimia nervosa had attained recovery by the 22-year follow-up; notably, the mean age of these participants was 47 years old. In addition, contrasting the widely held belief that illness chronicity impedes recovery, Bamford and Sly’s study [12] found that a longer duration of ED illness did not result in a lower quality of life. Therefore, the use of the age of 30 as an indicator of terminal illness in relation to EDs is unfounded and limits “older individual’s” pursuit of recovery.

EDs are *treatable illnesses*, even though the mortality rate is very high; this is synonymous with how many cancers and major depressive disorder are treatable diseases. While not everyone will survive their fight with these illnesses, it is important to provide the support for individuals to pursue the best course of treatment whilst also respecting their autonomy. Individuals with EDs are oftentimes ambivalent to receiving treatment: as Geppert states, “most patients with cancer accept the majority of treatments…in contrast, the AN patients in these cases adamantly denied the life-threatening nature of their disease and the potentially lifesaving treatments offered” [6, p. 37]. This refusal can be explained by growing research suggesting that biobehavioral syndromes alter the process of self-determination [13]. Similar to addiction, changes in brain function occur, such as alteration of the dopamine reward system, resulting in differences in mediation of pleasure, pursuit of rewarding stimuli, and habituation [13]. Given these variations, the disordered behavior becomes rewarding by providing structure, control, mastery, and avoidance of negative emotions [13, 14]. While the presence of a mental illness does not equate to one’s inability to make decisions, the egosyntonic nature of EDs—along with decreased functional cognitive skills such as poor insight, diminished cognitive flexibility, and decreased attention and concentration—limits the ability of patients with EDs to provide informed refusal of treatment [6, 15]. The literature [6] demonstrates that these cognitive functions improve when one’s nutritional needs (including weight restoration) are met. Consequently, it is essential that health care providers “reinforc[e] the ethical obligation to aggressively treat the [eating] disorder so that patients may regain the capacity to again make their own [rather than the ED’s] choices” [6, p. 37].

A second concern with Gaudiani’s article [1] is that there is limited information provided as to the degree and intentionality with which participants actively engaged in evidence-based treatment and whether the individual was at an appropriate level of care (e.g., inpatient versus partial hospitalization program versus outpatient) for their level of acuity. In Jessica’s case, she received outpatient treatment and had two brief stays in inpatient/residential treatment prior to leaving against medical advice [1]. Alyssa “had not completed a full residential eating disorder program”, “never fully restored weight”, and would not consent to further treatment recommendations [1, p. 9]. Individuals who refuse treatment, such as Alyssa, are described in literature to have as “a primary symptom of the disorder a pathological rejection of life-sustaining medical treatment” [6, p. 34]. Given the psychological, emotional, and physical difficulty associated with deciding to fight toward recovery from an ED, it is crucial that healthcare providers demonstrate passion and empathy as they encourage individuals in choosing to make the most out of treatment. Even when individuals are dismissive and/or uninterested in receiving higher levels of care, this is something that needs to be thoroughly discussed and explored [14]. Evidence-based treatment facilitates the opportunity for individuals with EDs to interrupt the learned, habituated, and automatic behaviors associated with the disorder.

The third concern raised is there is no clear indication that the patients’ comorbidities (e.g., depression, obsessive compulsive disorder [OCD], anxiety, suicidality) were appropriately treated. Ketamine is mentioned as being provided or offered to the patients, but there was no report of other commonly utilized treatments for these illnesses outside of medication, such as electroconvulsive therapy [16] or transcranial magnetic stimulation [17]. The literature [18] provides evidence that reducing levels of depression in people with AN improves their life satisfaction, and thus must be incorporated into treatment. This is part of the reason why higher levels of care are more advantageous when treating the entirety of the person: the disordered eating behavior and the comorbidities.

Within Gaudiani’s article [1], the ambivalence to engage in treatment and the impact of comorbid mental illness is evident in Aaron’s case. Though Aaron was admitted to intensive treatment a multitude of times, he is noted to have “had absolutely no motivation for recovery” [1, p. 3]. Since readiness to change is required for one to work toward obtaining and maintaining ED recovery, it is unlikely that Aaron would have been willing to actively partake in treatment. The proposal that Aaron’s refusal to eat was “less about ‘wanting to die’ than simply accepting that he could not live—he was not ‘attracted to life’” [1, p. 4] is reflective of the hopelessness associated with severe, untreated depression. The intensity of depression, OCD, and suicidality present combined with minimal active engagement in treatment also likely further exacerbated his “wish[es that] his AN would have already taken his life” [1, p. 3]. These comorbidities accentuate the necessity to provide treatment with a variety of interventions to ensure that the individual has the best opportunity to work toward recovery.

The fourth concern of Gaudiani’s article revolves around the physician’s assurance “that guardianship and forced treatment were likely now to be futile” for patient Jessica [1, p. 6]. There is limited evidence to substantiate this claim. Jessica’s story is somewhat convoluted: her parents had previously sought guardianship when she refused a higher level of care but lost the case for unclear reasons. Jessica had several instances of leaving treatment against medical advice—which was within her rights—and yet does not mean that her treatment was futile as it prolonged her life thus giving her an opportunity to choose to pursue recovery. Jessica experienced suicidality aligning with Joiner’s interpersonal theory of suicidal behavior which states that “the most dangerous form of suicidal desire is caused by the simultaneous presence of two interpersonal constructs—thwarted belongingness and perceived burdensomeness (and hopelessness about these states)” [19, p. 2]. These traits are evident within Jessica’s story: Jessica apologized for what she put her family through (perceived sense of burden, shame); she expressed that she hated her ED (self-blame, shame, self-hatred); Jessica “repeatedly told her family that she didn’t want to die, that she didn’t want to miss out on future time with her family, friends, and niece and nephew, but she just couldn’t continue to exist this way” (perceived burdensomeness, loss of social connectedness, hopelessness, helplessness, thwarted belongingness, physical pain, emotional/mental pain) [1, p. 6]. Furthermore, Joiner’s theory [19, p. 12] emphasizes that perceived burdensomeness includes two categories: “beliefs that the self is so flawed as to be a liability on others, and affectively-laden cognitions of self-hatred”—the latter of which includes low self-esteem; self-blame and shame; and agitated mental state, which are common symptoms of an ED [18, 20]. These negative thought patterns as well as acute/chronic physical illness due to the ramifications of eating disordered behavior further increases one’s sense of being a burden [6, 20].

Once thwarted belongingness and perceived burdensomeness are present, a person is more likely to desire suicide [19]. This desire for suicide is not in and of itself worrisome. According to Joiner, “the capability to engage in suicidal behavior is separate from the desire to engage in suicidal behavior” [19, p. 2]. The desire for suicide is quite commonly experienced by people with EDs and suicide does not immediately result, as evidenced by previous findings which state 37% of individuals with AN experience suicidal ideation [21] yet only 0.24% of individuals with AN complete suicide [4]. The problem, therein, lies when this person also has the *capability* to complete suicide or self-inflict death—which is exactly what MAID offers. Despite Jessica’s evident suicidality, she demonstrated clear ambivalence toward death: she “set multiple dates to use [MAID] over a couple of months and changed her mind as the date got closer” [1, p. 6]. While many people with EDs experience this suicidality, they do not have the capability to complete suicide; MAID provided Jessica with the capability to end her life.

## Conclusions

Gaudiani et al.’s [1] argument for MAID is that it provides control and compassion to the individual. No one chooses to have an ED, in the same way that no one chooses to have depression, cancer, a stroke, or diabetes. People have the right to choose the extent of treatment that they do (or do not) receive and the right to pursue hospice care if they decide to no longer engage in treatment. They have the right to choose whether they are resuscitated when experiencing cardiac arrest. They have the right to choose how extreme the measures are that are taken to prolong their life—this *is* a human right. Providing MAID for individuals struggling with EDs is wrong because it is an active step taken toward death, which some consider to be suicide, and is different than choosing to refuse other forms of treatment.

## Data Availability

Not applicable.

## Abbreviations

AN: Anorexia nervosa
ED: Eating disorder
EDs: Eating disorders
MAID: Medical aid in dying
OCD: Obsessive compulsive disorders
